# The 28S rRNA RT-qPCR assay for host depletion evaluation to enhance avian virus detection in Illumina and Nanopore sequencing

**DOI:** 10.3389/fmicb.2024.1328987

**Published:** 2024-01-31

**Authors:** Iryna V. Goraichuk, Mark Harden, Erica Spackman, David L. Suarez

**Affiliations:** ^1^Southeast Poultry Research Laboratory, U.S. National Poultry Research Center, Agriculture Research Service, U.S. Department of Agriculture, Athens, GA, United States; ^2^College of Veterinary Medicine, Tuskegee University, Tuskegee, AL, United States

**Keywords:** next-generation sequencing (NGS), Illumina, Nanopore, MinION, 28S, RNA virus, depletion

## Abstract

Abundant host and bacterial sequences can obscure the detection of less prevalent viruses in untargeted next-generation sequencing (NGS). Efficient removal of these non-targeted sequences is vital for accurate viral detection. This study presents a novel 28S ribosomal RNA (rRNA) RT-qPCR assay designed to assess the efficiency of avian rRNA depletion before conducting costly NGS for the detection of avian RNA viruses. The comprehensive evaluation of this 28S-test focuses on substituting DNase I with alternative DNases in our established depletion protocols and finetuning essential parameters for reliable host rRNA depletion. To validate the effectiveness of the 28S-test, we compared its performance with NGS results obtained from both Illumina and Nanopore sequencing platforms. This evaluation utilized swab samples from chickens infected with highly pathogenic avian influenza virus, subjected to established and modified depletion protocols. Both methods significantly reduced host rRNA levels, but using the alternative DNase had superior performance. Additionally, utilizing the 28S-test, we explored cost- and time-effective strategies, such as reduced probe concentrations and other alternative DNase usage, assessed the impact of filtration pre-treatment, and evaluated various experimental parameters to further optimize the depletion protocol. Our findings underscore the value of the 28S-test in optimizing depletion methods for advancing improvements in avian disease research through NGS.

## 1 Introduction

In recent years, untargeted next-generation sequencing (NGS) technology has been increasingly employed for the detection and characterization of RNA viruses, particularly in the context of avian diseases (Chen et al., 2013; Tang et al., 2015; Goraichuk et al., 2016, 2017, 2019, 2021a,b; Croville et al., 2018; Ferreri et al., 2019; Tal et al., 2019; Patzina-Mehling et al., 2020; Crossley et al., 2021; Chrzastek et al., 2022; Damir et al., 2023; Ip et al., 2023; Kariithi et al., 2023; Techera et al., 2023). Avian viruses, including highly pathogenic avian influenza virus (AIV) and Newcastle disease virus (NDV), pose significant threats to poultry populations and public health (Alexander, 1998; Suarez, 2017; Swayne et al., 2020). One of the biggest risks to the global poultry industry is the loss of animals and reduced egg production associated with infection by these RNA viruses. Whole-genome sequencing has become an important tool for the characterization of transmission and epidemiology of infectious diseases (Eyre, 2022). For example, the sequencing of AIV was instrumental in defining the multiple introductions of highly pathogenic AIV into North America in 2021–2022 (Alkie et al., 2022; Bevins et al., 2022; Caliendo et al., 2022; Engelsma et al., 2022; Günther et al., 2022) and tracking outbreaks (Rasmussen et al., 2023; Williams et al., 2023), farm-to-farm spread (Nagy et al., 2023; Youk et al., 2023), and spillovers to mammals (Agüero et al., 2023; Elsmo et al., 2023; Leguia et al., 2023; Nguyen et al., 2023; Puryear et al., 2023; Vreman et al., 2023). The diversity of poultry viruses is large, and concurrent infection with other infectious agents can complicate disease diagnosis. Therefore, the accurate and timely detection and characterization of avian viruses is crucial for effective disease control, surveillance, and management.

The typical NGS run is both cost and labor-intensive. Thus, a common aim of all NGS experiments is to maximize the yield of sequence reads of interest. The successful application of NGS for virus detection in samples faces a critical challenge—the presence of abundant host and bacterial sequences. These non-target sequences commonly include ribosomal RNA (rRNA), which can comprise up to 95% of total reads depending on the sample type (Morlan et al., 2012; Fauver et al., 2019). These sequences can overshadow the viral genetic material, making it difficult to detect and characterize the less prevalent viruses of interest (Morlan et al., 2012; Shi et al., 2022). The removal of the most abundant host-specific rRNA (18S, 28S, mitochondrial) and bacterial rRNA (16S, 23S) is a fundamental step in addressing this issue, as it can substantially improve the sensitivity and accuracy of viral detection in NGS data.

Currently, depletion methods often involve enzymatic treatment (Allander et al., 2001; Kapoor et al., 2008; Victoria et al., 2008; Bal et al., 2018; Marotz et al., 2018; Oechslin et al., 2018; Oristo et al., 2018; Nelson et al., 2019; Shi et al., 2019; Amar et al., 2021; Bruggeling et al., 2021; Gan et al., 2021), probe hybridization-based methods (Metsky et al., 2017; Stark et al., 2019; Zeng et al., 2020; Gu et al., 2021; Parris et al., 2022), DNA-intercalating dyes (Nocker et al., 2009; Fittipaldi et al., 2012), cell lysis (Hasan et al., 2016; Charalampous et al., 2019; Yeoh, 2021), size selection (Ng et al., 2009; Hall et al., 2014; Kohl et al., 2015; Liang and Bushman, 2021; Wang et al., 2021), and targeted enrichment (Lee et al., 2017; Imai et al., 2018; Freed et al., 2020; Galli et al., 2022; Singh, 2022). While these methods are effective to some extent, their efficiency can vary, and the choice of enzymes, filter size, etc., can significantly impact NGS outcomes and add to the cost of testing each sample. Thus, there is a need for a systematic evaluation and optimization of depletion methodologies to enhance the detection of avian viruses via NGS. In our prior studies, we developed a targeted RNase H-based depletion approach (Parris et al., 2022; Bakre et al., 2023) to reduce the abundance of host and bacterial reads using DNA probes designed to target chicken 18S and 28S rRNA, specific chicken mitochondrial RNA, and select 16S and 23S bacterial rRNA (O'Flaherty et al., 2018; Wille et al., 2018). Nonetheless, we consistently engage in an optimization process to ensure that our protocols are tuned for improved viral detection capabilities through increased sequencing depth and genome breadth of coverage for various avian viral pathogens of interest. Therefore, a reliable cost-effective quantitative polymerase chain reaction (qPCR) assay was sought as an alternative to the cost-intensive NGS in the further optimization of the depletion methods for effective reduction of host rRNA abundance in the analyzed samples, ultimately heightening sequencing depth and breadth of coverage of the target viral pathogens in these specimens.

The objective of this work was to develop a superior method to deplete host rRNA before NGS library preparation and we also introduce a novel 28S rRNA reverse transcription qPCR (RT-qPCR) assay. This assay was designed to assess the efficiency of depletion methods before conducting costly NGS for avian virus detection. This comprehensive evaluation focuses on evaluating different DNAses in our established RNase H-based depletion protocol (Parris et al., 2022; Bakre et al., 2023), fine-tuning essential parameters for reliable host rRNA depletion, and exploring other cost- and time-effective strategies. The significance of this work lies in the 28S-test potential to facilitate the further optimization of different depletion protocols to improve the accuracy and sensitivity of avian virus detection via NGS, ultimately contributing to better disease management and our understanding of avian virus abundance.

## 2 Materials and methods

### 2.1 Samples

To validate the performance of the 28S rRNA RT-qPCR assay, we compared its results to Nanopore and Illumina NGS data. Five oropharyngeal (OP) and cloacal (CL) swabs were collected from 5 six-week-old specific-pathogen-free (SPF) white leghorn chickens (*Gallus domesticus*; obtained from the Southeast Poultry Research Laboratory in-house flocks, Athens, GA, United States) after they succumbed to infection with a high dose of 6 log_10_EID_50_ per bird in 0.1 mL of highly pathogenic H5N1 AIV A/turkey/Indiana/22-003707-003/2022 in the animal BSL-3E facilities. Chickens had *ad libitum* access to food and water throughout the experiment. The swabs were immediately deposited into sterile cryovials containing 2 mL of brain heart infusion (BHI) transport media and were stored at 4°C. Additionally, OP and CL swabs obtained from four euthanized SPF chickens (n = 8) were spiked with low pathogenic LaSota Newcastle Disease Virus (NDV) and were used to mimic field samples for the optimization of 28S rRNA RT-qPCR.

### 2.2 Depletion methods

**Figure 1:**
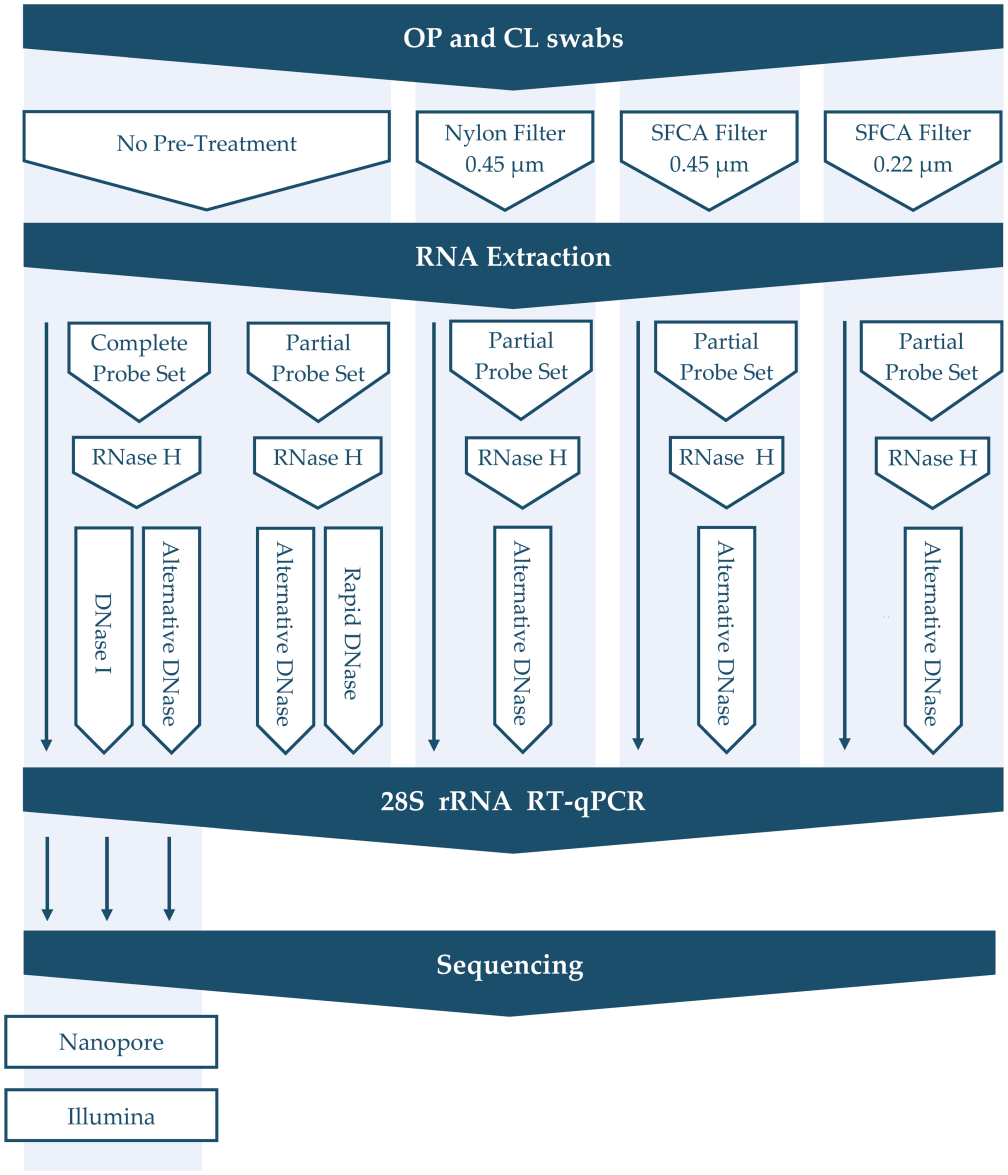
Overview of depletion methods applied in this study.

Aliquots of RNA extracts from the samples described above were treated with different depletion methods designed to remove abundant non-target host and bacterial sequences (Figure 1). These treatments included filtration pre-treatment before nucleic acid extraction (0.45 μm Nylon, 0.45 μm Nalgene surfactant-free cellulose acetate (SFCA), and 0.22 μm Nalgene SFCA Syringe Filters, Thermo Scientific, United States) and RNase H probe hybridization followed by different DNase digestions. Our main focus was to refine our established rRNA depletion method to enhance its capability to selectively remove specific rRNA (18S, 28S, mitochondrial) and bacterial rRNA (16S, 23S). This method was compared to modified protocols in which DNase I (NEB, United States) was substituted with the alternative DNase (TURBO DNA-free kit; Invitrogen, United States) or rapid DNase (ezDNase, Invitrogen, United States), which were all done in parallel. Additionally, a partial probe set (CK28s rRNA4 and CK28s rRNA5) of varying concentrations was tested in conjunction with the alternative DNase digestion to selectively deplete chicken 28S rRNA (Parris et al., 2022; Bakre et al., 2023). Briefly, 12-μL aliquots of RNA (with and without filtration pre-treatment) were hybridized with DNA probes by incubating at 95°C for 5 min, cooling gradually (0.1°C/s) to 22°C, and incubating for an additional 5 min at 22°C. RNA–DNA hybrids were then degraded by incubating with RNase H at 37°C for 30 min, and either DNase I, alternative DNase, or rapid DNase were used for further digestion (30, 30, or 2 min, correspondently) to remove excess DNA probes. The alternative DNase digestion was stopped by adding 0.2 volumes of DNase Inactivation Reagent as per the manufacturer’s recommendations. DNase digestion time (30, 45, 60 min), repeated RNase H degradation, as well as concentration of probes were also tested. Finally, RNA was purified with the AMPure RNAClean XP beads (Beckman Coulter, United States) at 2.2 volume. Untreated controls were included for each sample to provide a baseline reference.

### 2.3 Primer and probe design

We designed the 28S rRNA RT-qPCR assay for the performance evaluation of different depletion methods before NGS sequencing. After testing different sets of primers and probes targeting host 28S rRNA, the final set of forward primer 28S+3894 (5′-GTCGGCTCTTCCTATCATTGTG-3′), reverse primer 28S-4026 (5′-CGCAACAACACATCATCAGTAGG-3′) and probe 28S+3198 (5′-GCAGAATTCACCAAGCGTTGGATTGTTCACC-3′ FAM reporter dye with Zen/Black Hole Quencher 1; Integrated DNA Technologies, Iowa City, IA) was selected. We then optimized the annealing temperature RT-qPCR and evaluated the primer/probe concentration and ratio to attain optimal conditions.

The RT-qPCR 28S-test was performed using the AgPath-ID One-Step RT-PCR kit (Applied Biosystems, United States) in 25-μL reaction volumes comprised of 2 μL of total RNA, 12.5 μL of 2 × RT-PCR buffer, 0.5 μL of the forward and reverse primers (20 pmol/μL), 0.5 μL of the probe (6 pmol/μL), 1 μL of AgPath Enzyme Mix (Ambion, United States), and sterile nuclease-free water. The test included an initial RT step (10 min at 45°C and 10 min at 95°C) and PCR steps of 40 cycles (10 s at 94°C, 30 s at 57°C, and 10 s at 72°C). All RT-qPCR tests were performed on a QuantStudio 5 real-time PCR system (Applied Biosystems, United States).

Additionally, viral RNA preservation after different depletion methods was evaluated by the NVSL AIV matrix gene (M-test) RT-qPCR with the forward primer M+25 (5′-AGATGAGTCTTCTAACCGAGGTCG-3′), two reverse primers M-124 2002 (5′-TGCAAAAACATCTTCAAGTCTCTG-3′), M-124p 2009 (5′-TGCAAAGACACTTTCCAGTCTCTG-3′) and probe M+64 (5′-TCAGGCCCCCTCAAAGCCGA-3′) and the USDA-validated NDV M-test RT-qPCR with the forward primer M+4100 (5′-AGTGATGTGCTCGGACCTTC-3′), reverse primer M-4220 (5′-CCTGAGGAGAGGCATTTGCTA-3′) and probe M+4169 (5′-TTCTCTAGCAGTGGGACAGCCTGC-3′), as previously described (Spackman et al., 2002; Wise et al., 2004).

### 2.4 Library preparation and sequencing

To assess the effectiveness of our RT-qPCR 28S-test, we compared its performance to the results of long-read Nanopore and short-read Illumina sequencing. For this, 5 untreated RNA extracts from swab samples collected from AIV-infected birds were compared to the same extracts but treated with our custom depletion protocol using DNase I and the alternative DNase. Treated and untreated RNA extracts from each sample were amplified via sequence-independent, single-primer amplification (SISPA) as described previously (Chrzastek et al., 2017). Briefly, first-strand cDNA was synthesized using 100 pmol of K-8 N primer (5′-GACCATCTAGCGACCTCCACNNNNNNNN-3′) with the SuperScript IV First-Strand System (Invitrogen, United States) following the manufacturer’s instructions. Second-strand synthesis was performed using the DNA Polymerase I, Large (Klenow) Fragment (NEB Inc., United States) with 10 pmol of K-8 N primer and 10 μM dNTPs and resulting dsDNA was bead purified using the AMPure XP beads (Beckman Coulter, United States). Amplification of cDNA was performed using the Phusion High-Fidelity PCR Kit (NEB Inc., United States) with the 10 μM of K primer (5′-GACCATCTAGCGACCTCCAC-3′) under the following conditions: 98°C for 30 s, followed by 35 cycles of 98°C for 10 s, 55°C for 30 s, and 72°C for 1 min, with a final extension at 72°C for 10 min. After the SISPA amplification step, amplicons were bead-purified in a 1:1.8 sample volume to bead volume ratio and quantified using the Qubit 1X dsDNA High Sensitivity Assay Kit (Invitrogen, United States), followed by sample library preparation for Nanopore and Illumina sequencing libraries.

The Nanopore sequencing libraries were prepared using the Native Barcoding Kit 24 V14 (SQK-NBD114.24, Oxford Nanopore Technologies, England) following the manufacturer’s instructions. The final library was quantified using a High Sensitivity D5000 Screen Tape on a 4150 TapeStation (Agilent Technologies, United States). We then sequenced 20 fmol of the prepared library on a single R10.4.1 flow cell (FLO-MIN114, Oxford Nanopore Technologies, England) using a MinION Mk1C instrument with the MinKNOW 23.04.8 software. Sequencing was run until all pores of the flow cell were exhausted (~48 h).

The Illumina libraries were prepared using the Illumina DNA Prep (Illumina, United States) according to the manufacturer’s recommendations. After quantification using the Qubit 1X dsDNA High Sensitivity Assay Kit (Invitrogen, United States) and High Sensitivity D5000 Screen Tape (Agilent Technologies, United States), the libraries were pooled (4 nM, 10 μL each), spiked with a control library (5% PhiX library v3), diluted to 12 pM final concentration and sequenced (paired-end; 2 × 300 bp) using the 600-cycle MiSeq Reagent Kit v3 (Illumina, United States) on an Illumina MiSeq instrument.

### 2.5 Data analysis

After the sequencing run, Nanopore raw Pod5 files were basecalled (high-accuracy, 400kbs) to generate FastQ files, that were further demultiplexed and trimmed using Guppy 6.5.7 within the MinKNOW 23.04.8 software on a MinION Mk1C instrument. Reads with a minimum quality of 9 were considered for further analysis.

The Illumina raw sequencing data was pre-processed within the Galaxy platform, as described previously (Dimitrov et al., 2017). Raw sequence reads were quality assessed using FastQC v0.63 (Andrews, 2010) residual adaptor sequence and low-quality bases were trimmed using Cutadapt v1.16.6 (Martin, 2011). After control library reads were removed using the Burrows-Wheeler alignment tool (BWA-MEM; Li and Durbin, 2009), forward and reverse reads were merged using PEAR v.0.9.6.1 (Zhang et al., 2014).

The host (*Gallus gallus*) reads were removed from the pre-processed Nanopore and Illumina reads using a BWA-MEM tool. The remaining unmapped reads were further used for taxonomical classification by Kraken2 v2.0.8 using the PlusPF database (Wood and Salzberg, 2014; Wood et al., 2019) and AIV genome consensus assembly by BWA-MEM mapping with reference genome A/Turkey/Indiana/22–003707-003/2022 (H5N1; GenBank accession numbers OQ965225 to OQ965232) within the Galaxy platform. The Kraken2 classified reads were further processed with Bracken v2.5 (Lu et al., 2017) to estimate relative abundance at the family level. Individual Bracken taxonomy tables for each treatment were merged with the “combine_bracken_outputs.py” python script. The merged Bracken data was processed with the R application “bracken_plot” (Vill, 2023) to determine and visualize the top 10 taxa with the greatest median relative abundances.

### 2.6 Statistical analysis

GraphPad Prism 9.3.1 was used for data representation and statistical analysis. The One-way ANOVA followed by Tukey’s multiple comparisons test was utilized to compare the relative difference of cycle threshold (Ct) values among different depletion methods with untreated control samples. For statistical purposes, all swab samples with negative RT-qPCR results were assigned a Ct value of 40. The value of *p* < 0.05 was considered statistically significant.

## 3 Results

### 3.1 RT-qPCR 28S-test design and optimization

In our efforts to optimize the RT-qPCR 28S-test conditions, we focused on refining the primer annealing temperature, primer/probe concentration, and selecting the most suitable primer pair set. To achieve this, we designed four pairs of sequence-specific primers targeting host 28S rRNA based on sequences available in the NCBI database. These primers were carefully designed to meet specific criteria. They were relatively short, ranging from 20 to 25 base pairs in length. We avoided the presence of three or more consecutive nucleotides of the same type (e.g., AAA or GGG) in any of the primers. Additionally, we ensured that each primer had a C or G at the last nucleotide position at either or both ends, and their GC content ranged from 45 to 50%.

Serial dilutions (1:10 dilution) of total RNA extracted from OP and CL swabs collected from SPF birds were used to test the performance of different primer/probe sets and their concentration. To identify the optimal annealing temperature for each primer pair, we conducted gradient RT-qPCR. After a series of tests, we determined that the best-performing primer/probe set was 28S+3894, 28S-4026, and 28S+3918. We further refined the assay by selecting the optimal primer/probe concentration and annealing temperature (data not shown). after testing different concentrations, 10 pmol of each primer and 3 pmol of probe per reaction were chosen as optimal, at an annealing temperature of 57°C.

### 3.2 Next-generation sequencing

**Figure 2:**
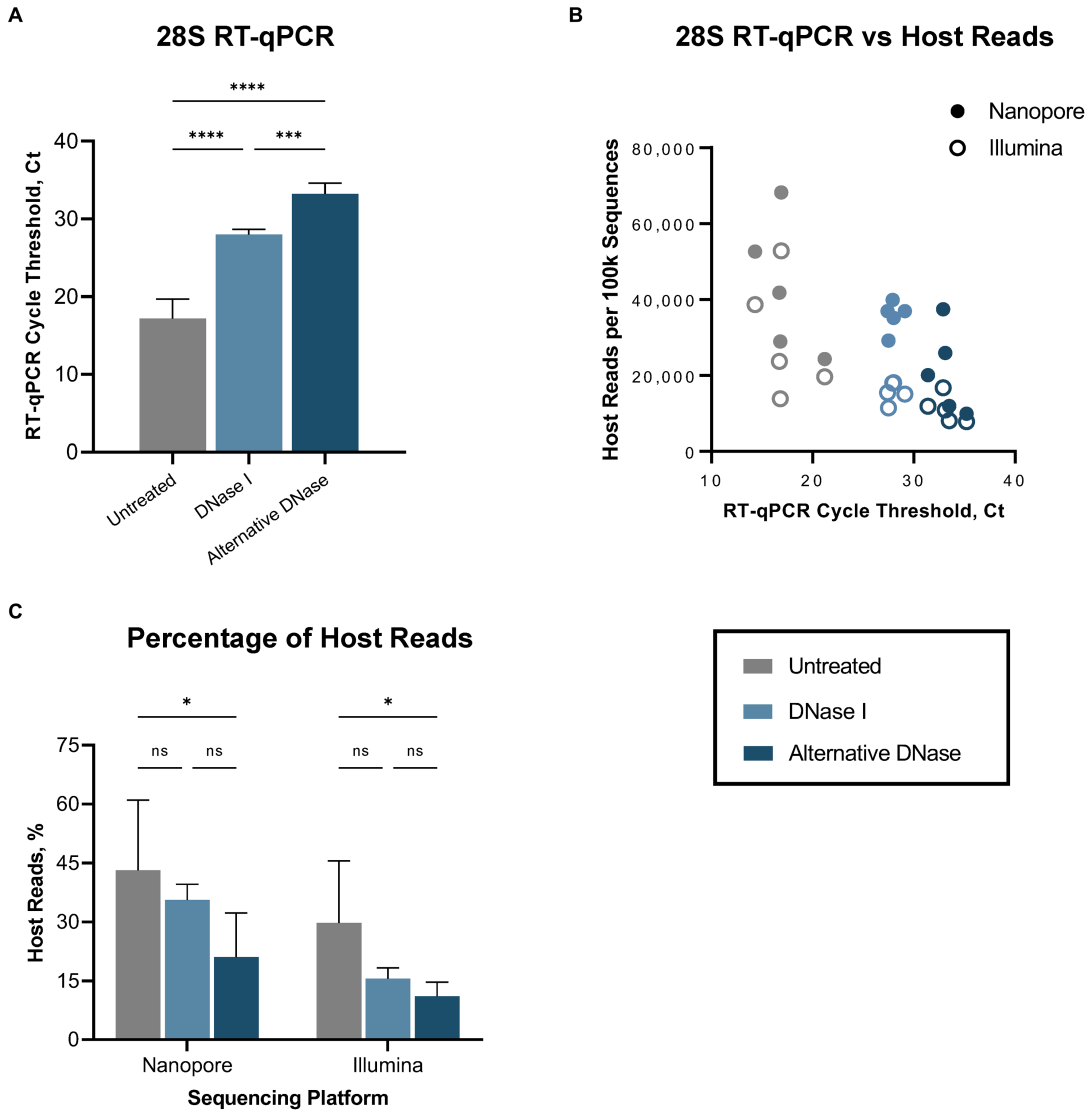
Host (*Gallus gallus*) reads depletion outcome. **(A)** Average cycle threshold (Ct) values of 28S rRNA RT-qPCR assay in different depletion methods. **(B)** Relationship between 28S rRNA RT-qPCR Ct values and number of sequenced host-specific reads. **(C)** The average percentage of reads mapping to the host genome in different depletion treatments obtained on Nanopore and Illumina sequencing platforms. One-way ANOVA with Tukey’s multiple comparison analysis was used to evaluate the significance between different depletion methods. A value of *p*  <  0.05 was considered to be significant. ^*^p < 0.05; ^**^p < 0.01; ^***^p < 0.001; ^****^p < 0.0001.

First, we evaluated the performance of the RT-qPCR 28S-test comparing our established RNase H-based rRNA depletion assay (Parris et al., 2022; Bakre et al., 2023) and a modified protocol in which evaluating alternative DNases. This assessment was performed on five swabs collected from SPF chickens experimentally infected with the highly pathogenic H5N1 AIV. These samples were subjected to the established and modified depletion methods utilizing our complete set of custom probes, which selectively depletes host-specific rRNA (18S, 28S, mitochondrial) and bacterial rRNA (16S, 23S). Next, we prepared Nanopore and Illumina sequencing libraries for three sets of samples: untreated, samples after depletion with DNase I, and samples after depletion with the alternative DNase. Both depletion treatments substantially (*p* < 0.0001) increased the average Ct values in the RT-qPCR 28S-test when compared to untreated control samples (Figure 2A; Supplementary Table 1). However, significantly higher Ct values (*p* < 0.001) were observed in samples treated with the alternative DNase compared to samples treated with DNase I, indicating a more effective reduction in host-specific rRNA. Notably, these results corresponded to the NGS outcome on both Nanopore and Illumina platforms (Figure 2B). In swabs with higher Ct values a reduced number of host-specific reads per 100 k obtained was observed on both sequencing platforms. The trend between depletion methods in the elevation of the average Ct values had an inverse correlation with the decrease in the average percentage of host reads on both sequencing platforms (Figure 2C; Supplementary Table 1). Although both depletion treatments led to a decrease in the average percentage of host-specific reads, a statistically significant reduction (*p* < 0.05) was only evident in samples treated with the alternative DNase.

**Figure 3:**
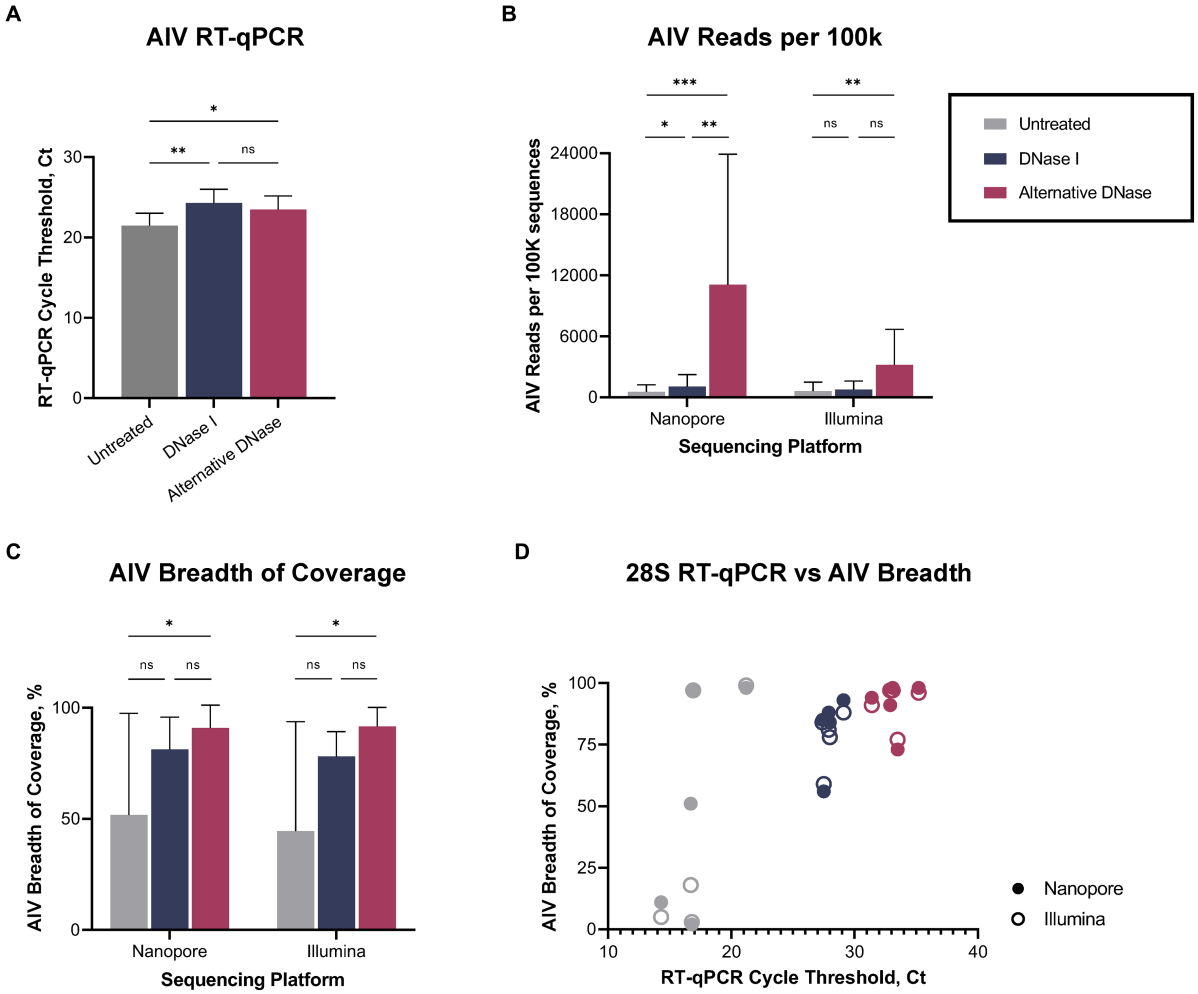
Viral reads recovery after DNase I and alternative DNase treatments. **(A)** Average cycle threshold (Ct) values of avian influenza virus (AIV) M gene RT-qPCR assay in different depletion methods. **(B)** Average number of AIV reads per 100 k sequences. **(C)** Average percentage of the breadth of AIV genome coverage. **(D)** Relationship between 28S rRNA RT-qPCR Ct value and percentage of the breadth of AIV genome coverage in different depletion treatments obtained on Nanopore and Illumina sequencing platforms. One-way ANOVA with Tukey’s multiple comparison analysis was used to evaluate the significance between different depletion methods. A value of *p*  <  0.05 was considered to be significant. ^*^p < 0.05; ^**^p < 0.01; ^***^p < 0.001; ^****^p < 0.0001.

Regardless of the depletion assay used, both DNase treatments led to a reduction not only in host-specific but also bacterial reads (Supplementary Figure 1), consequently enabling a notable increase in the number of sequenced viral reads (Supplementary Table 1). The changes in bacterial abundance correlated with the bacterial rRNA (16S, 23S) that were targeted by hybridization probes during depletion. Thus, we observed the reduction of reads belonging to families that were targeted during depletion (*Lactobacillaceae*, *Oscillospiraceae*, *Yersiniaceae, except Enterobacteriaceae*) and a consequent rise of untargeted reads (*Orthomyxoviridae*, *Hominidae*, *Enterococcaceae*, *Staphylococcaceae*, *Streptomycetaceae*, *Prevotellaceae*) compared to untreated samples (Supplementary Figure 2). Notably, while DNase I and alternative DNase depletion treatments both slightly reduced viral RNA (*p* < 0.01 and *p* < 0.05, correspondently), represented by elevated Ct values in the AIV M-test (Figure 3A), they provided increase in the relative abundances of sequenced reads belonging to family *Orthomyxoviridae* (Supplementary Figure 2). The alternative DNase outperformed DNase I, resulting in higher Ct values in the 28S-test, lower Ct values in the AIV M-test, and a higher increase of sequenced viral reads. Specifically, in samples treated with the alternative DNase, viral reads constituted 7.7% and 3.1% of the total reads, whereas only 0.4% and 0.6% of viral reads were presented in untreated samples when sequenced on Nanopore and Illumina platforms, respectively. Moreover, a substantial 7-fold and 5-fold increase in the average number of viral reads per 100 k sequences was observed in samples treated with the alternative DNase compared to those treated with DNase I and subsequently sequenced on Nanopore and Illumina platforms, correspondently (Figure 3B). This elevation in sequenced viral reads subsequently contributed to the improved breadth of the AIV genome after consensus assembly (Figure 3C; Supplementary Table 1). It is noteworthy that, although there was an increase in the average percentage of AIV genome breadth in samples subject to both depletion assays, only the use of the alternative DNase resulted in a statistically significant (*p* < 0.05) enhancement in viral genome breadth (Figure 3C). In fact, both Nanopore and Illumina platforms yielded an average AIV genome breadth exceeding 90% after the alternative DNase treatment. Furthermore, regardless of the type of DNase used, the depletion of host-specific and bacterial rRNA correlated with the enhanced breadth of viral genome coverage obtained on both sequencing platforms (Figure 3D). Therefore, the results of the developed RT-qPCR 28S-test were confirmed by positive correlation with the sequencing outcome, demonstrating its potential to facilitate further optimizations of host rRNA depletion for improved detection of avian pathogens.

### 3.3 Depletion treatments

#### 3.3.1 DNase digestion

**Figure 4:**
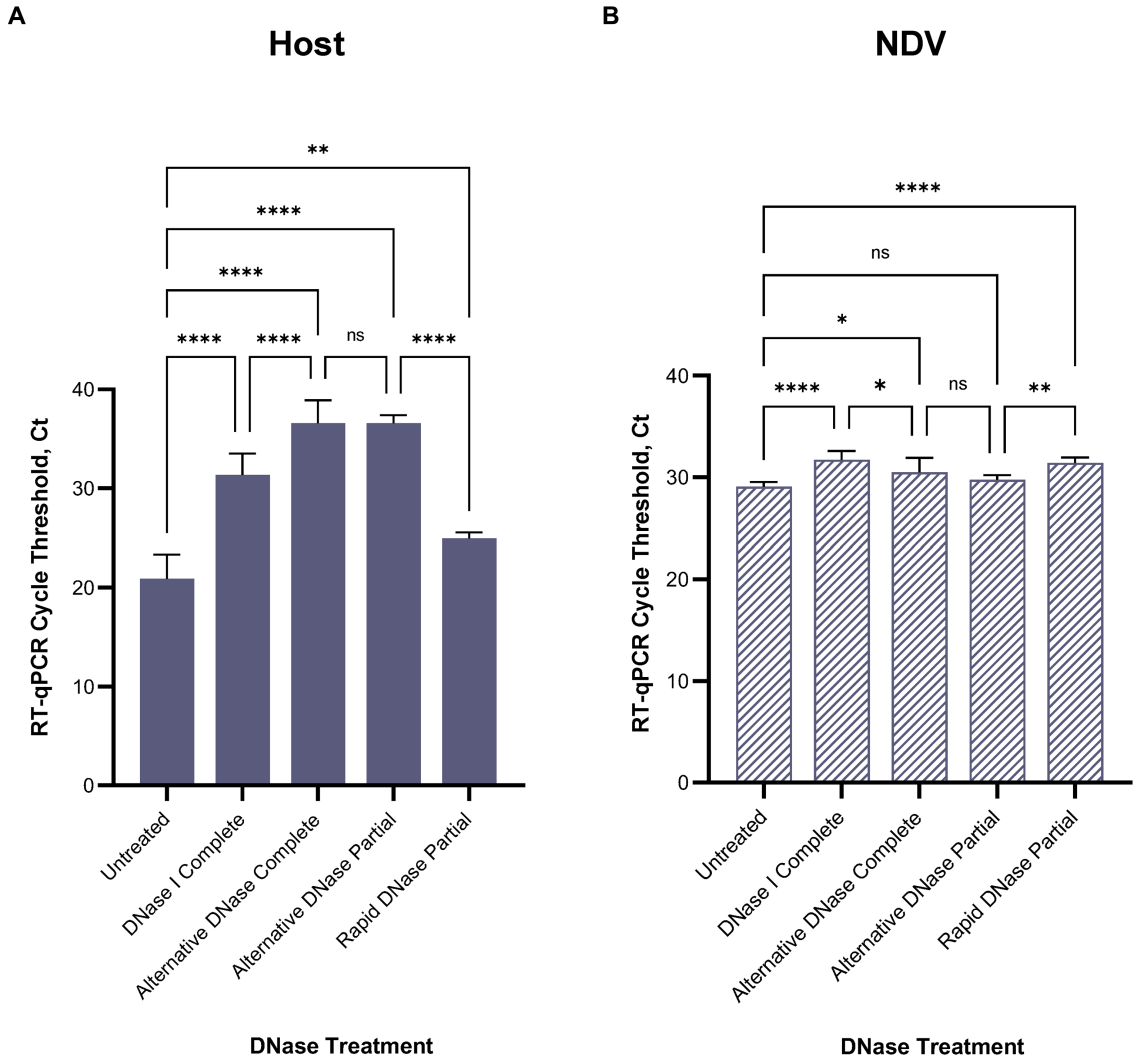
Average **(A)** chicken 28S rRNA and **(B)** Newcastle disease virus (NDV) M gene RT-qPCR cycle threshold (Ct) values in each depletion treatment. All treatments significantly reduced host rRNA, which was represented by elevated Ct values compared to untreated control samples. One-way ANOVA with Tukey’s multiple comparison analysis was used to evaluate the significance between Ct values in different depletion methods. A value of *p*  <  0.05 was considered to be significant. ^*^p < 0.05; ^**^p < 0.01; ^***^p < 0.001; ^****^p < 0.0001.

After confirming RT-qPCR 28S-test results with the NGS results using swabs collected from birds infected with highly pathogenic H5N1 AIV, we repeated the comparison of substituting DNase I with the alternative DNase in our depletion protocol using swab samples collected from SPF chickens and spiked with the low pathogenic LaSota NDV. The RT-qPCR NDV M-test was conducted to assess the preservation of viral RNA after the depletion treatments. Consistent with our prior observations, the results indicated that both DNase I and alternative DNase treatments significantly (*p* < 0.0001) reduced host rRNA levels compared to untreated controls (Figure 4A), as evidenced by the elevated Ct values in RT-qPCR 28S-test. Depletion treatment with the alternative DNase resulted in a more substantial increase in RT-qPCR threshold values (ranging from 33.5 to 40 Ct), with an average increase of 15.7 Ct compared to untreated control samples (17.8–23.6 Ct). In contrast, depletion with the use of DNase I increased threshold values only by an average of 10.5 Ct (ranging from 28.5 to 34.2 Ct). Importantly, the threshold values in the NDV M-test exhibited only a modest increase with the alternative DNase treatment resulting in a 1.4 Ct increase (*p* < 0.05), while DNase I resulted in a more substantial increase of a 2.6 Ct (*p* < 0.0001) compared to untreated samples (Figure 4B). These findings suggest that both depletion methods effectively reduce host rRNA levels, with the alternative DNase showing a more pronounced effect.

For further evaluation of depletion methods and performance of RT-qPCR 28S-test, we selected the alternative DNase based on its superior performance in reducing host rRNA levels. Additionally, to reduce the price per reaction during testing, we compared our RNase H-based depletion protocol using a complete set of probes with a partial set of probes (chicken 28S rRNA4 and rRNA5). To facilitate this, the amplification region of the 28S-test was within the targeted region for depletion with the reduced set of probes. As anticipated, depletion with the partial set of probes performed comparably to depletion with the complete set, and there were no significant differences between these two sets in the RT-qPCR 28S-test (Figures 4A,B). The average cycle threshold values were elevated by 15.7 Ct (ranging from 35.4 to 37.4 Ct) compared to untreated control samples (Figure 4A). Similarly, in the NDV M-test, there was only a minor increase of 0.7 Ct compared to untreated controls (Figure 4B).

Furthermore, we evaluated the feasibility of substituting DNase I and the alternative DNase with rapid DNase to reduce preparation time. DNase I and the alternative DNase digestion typically require 30-min incubation at 37°C, whereas rapid DNase requires only 2 min. It is important to note that despite the elevation of average Ct values compared to untreated samples, depletion with rapid DNase resulted in a significantly lower reduction (*p* < 0.0001) of host rRNA compared to the alternative DNase (Figure 4A). The average Ct values in samples after rapid DNAse treatment were 11.6 Ct lower (ranging from 24.3 to 26.1 Ct) than those in samples treated with the alternative DNase.

#### 3.3.2 Extraction pre-treatment

**Figure 5:**
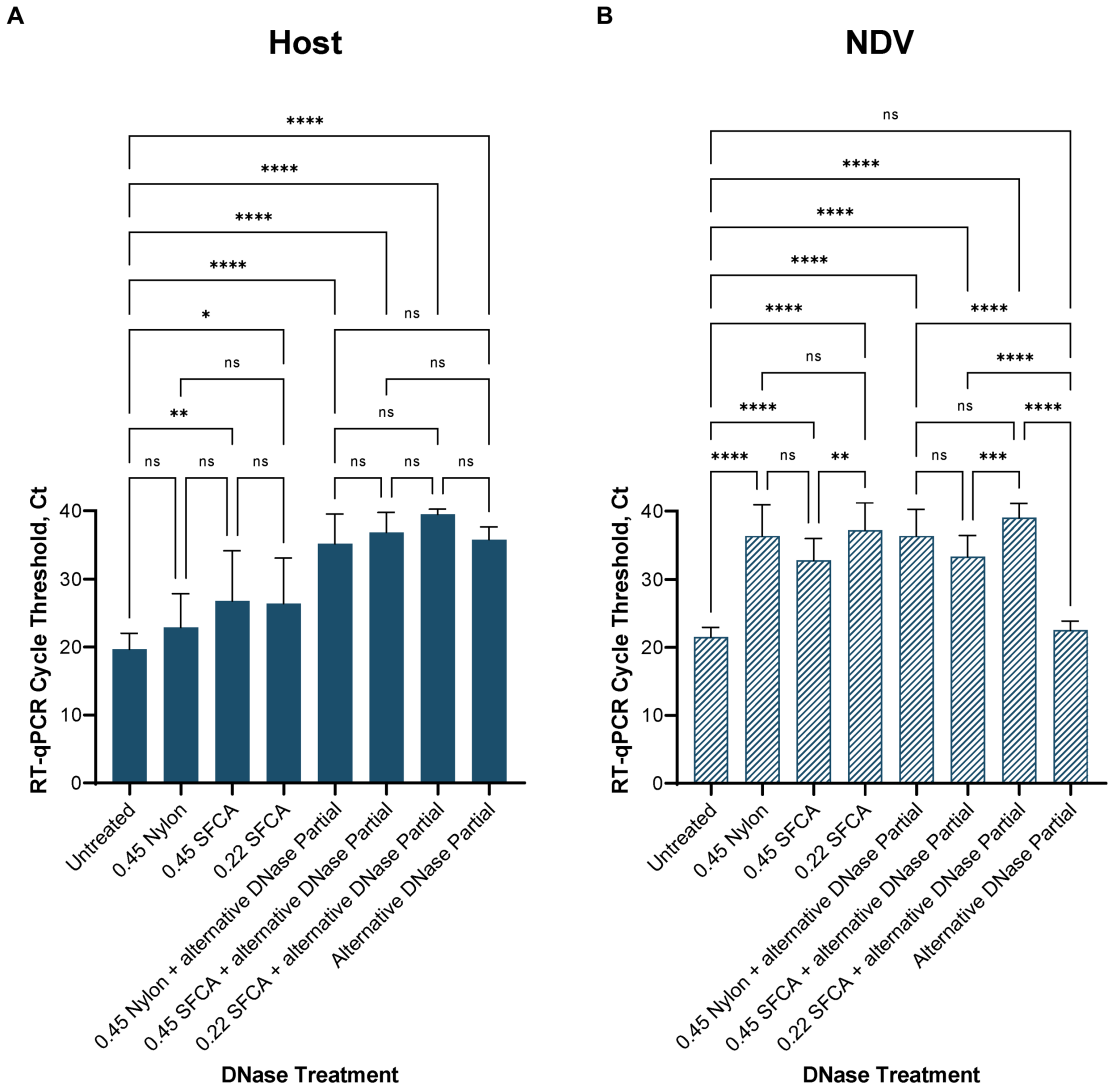
Average **(A)** chicken rRNA 28S and **(B)** Newcastle disease virus (NDV) M gene RT-qPCR cycle threshold (Ct) values in the alternative DNase depletion with and without filtration pre-treatment. Without subsequent alternative DNase treatment, only filtration with cellulose acetate syringe filters (SFCA) before extraction significantly reduced host rRNA. Filtration together with the alternative DNase depletion reduced host rRNA significantly better than filtration alone. However, regardless of whether post-extraction depletion treatment was applied or not, all filters significantly reduced viral RNA. One-way ANOVA with Tukey’s multiple comparison analysis was used to evaluate the significance between Ct values in different depletion methods. A value of *p*  <  0.05 was considered to be significant. ^*^p < 0.05; ^**^p < 0.01; ^***^p < 0.001; ^****^p < 0.0001.

We evaluated the impact of filtration pre-treatment using different types of syringe filters (0.45 μm Nylon, 0.45 μm Nalgene SFCA, and 0.22 μm Nalgene SFCA) prior to nucleic acid extraction. We assessed the effects of filtration methods independently and in combination with our depletion protocol using the alternative DNase. When evaluating the three different filters compared to each other, we observed no significant differences in the reduction of 28S chicken rRNA, regardless of whether post-extraction depletion treatment was applied or not (Figure 5A). However, when compared to untreated samples, only the filtration with cellulose acetate filters significantly reduced host rRNA levels, when no subsequent deletion was applied. Specifically, the 0.45 μm Nalgene SFCA filter led to a reduction with *p* < 0.001, and the 0.22 μm Nalgene SFCA filter resulted in a reduction with *p* < 0.05 (Figure 5A). Still, these reductions were observed to be lower than the reductions achieved when the filters were used in combination with the alternative DNase treatment. All filtration pre-treatments combined with the depletion treatment significantly reduced (*p* < 0.0001) host rRNA. Though, these pre-treatments provided no significant difference with the alternative DNase alone.

Adversely, viral RNA was significantly reduced (*p* < 0.0001) by filtration, regardless of filter type used and whether depletion treatment was applied or not (Figure 5B) when compared to untreated samples and samples after the alternative DNase treatment. This suggests that filtration pre-treatment had a detrimental effect on viral RNA, leading to a substantial reduction in its quantity. In contrast, the depletion using the alternative DNase without any extraction pre-treatment not only significantly (*p* < 0.0001) reduced 28S host rRNA, as intended, but at the same time did not significantly reduce the amount of viral RNA.

#### 3.3.3 Optimization of depletion protocol

In our study, we evaluated the impact of various experimental parameters on the reduction of host rRNA and preservation of viral RNA. Specifically, we examined the effect of extending the alternative DNase digestion time to 45 and 60 min, explored different concentrations of the partial probe set, and conducted repeated RNase H treatments. Extending the alternative DNase digestion time beyond the recommended 30 min did not significantly impact the reduction of 28S rRNA or viral RNA levels (Supplementary Figure 3A). Comparison of different partial probe set concentrations revealed a common trend of reduced Ct values or RT-qPCR targeting 28S rRNA when probe concentrations decreased (Supplementary Figure 3B). Furthermore, our RNase H-based depletion with a partial probe set yielded a significant reduction in host rRNA levels (*p* < 0.0001) across all concentrations tested, without affecting viral Ct values. When comparing repeated RNase H treatments to improve degradation of probe-bound rRNAs, one and three rounds of RNase H treatment resulted in a significant reduction in host rRNA levels compared to untreated samples (*p* < 0.0001 and *p* < 0.05, respectively). In contrast, depletion without RNase H treatment or with two RNase H treatments did not yield any statistically significant difference (Supplementary Figure 3C).

## 4 Discussion

In this study, we conducted a comprehensive evaluation of the newly developed 28S rRNA RT-qPCR as a tool to facilitate the optimization of host depletion methods to enhance avian virus detection via next-generation sequencing. Our approach involved a systematic assessment of this 28S-test in conjunction with various depletion protocols, with a particular focus on substituting DNase I with the alternative DNase in our established RNase H-based rRNA depletion protocol (Parris et al., 2022; Bakre et al., 2023). To ensure reliable detection of avian 28S rRNA, we fine-tuned primer annealing temperatures, optimized primer/probe concentrations, and selected the most suitable primer pair set. Our primer design adhered to specific criteria, including short lengths, nucleotide diversity, and a balanced GC content. Through rigorous testing, we identified the optimal primer/probe set as 28S+3894/-4026/+3918 and determined their ideal concentrations and annealing temperature.

It is known that commonly used conventional DNase I, which was first characterized more than a half-century ago, is salt-sensitive, has a poor affinity for DNA, and inefficiently cleaves DNA of low concentration (Kunitz, 1950; ThermoFisher, 2012). In addition, DNase I is purified from bovine pancreas, one of the richest natural sources of RNase A, which can be crucial when working with RNA viruses. The alternative DNase is an engineered version of wild-type DNase I with 350% greater catalytic efficiency and a markedly higher affinity for DNA, making it more effective in removing trace quantities of DNA contamination. The alternative DNase is also capable of maintaining up to 50-fold greater activity than DNase I in solutions at physiological salt concentrations and is RNase-free by nature (ThermoFisher, 2018). Therefore, our initial RT-qPCR 28S-test evaluation was performed using the Nanopore and Illumina sequencing instruments with results comparing untreated samples with the samples that underwent our custom depletion assay with DNase I, and a modified protocol using the alternative DNase. For this evaluation, we employed clinical swabs from SPF chickens infected with a highly pathogenic avian influenza virus A/turkey/Indiana/22–003707-003/2022 (H5N1). Both depletion methods significantly increased the average Ct values in the RT-qPCR 28S-test, indicating substantial host-specific rRNA reduction. Notably, substituting DNase I with the alternative DNase yielded significantly improved results, as evidenced by even a further elevation of Ct values in the 28S-test, signifying a more pronounced reduction in host-specific rRNA, and a lower Ct value elevation in the AIV M-test, indicating the preservation of viral RNA integrity. These improvements were consistent with NGS outcomes on both Nanopore and Illumina platforms. It is important to highlight that while both depletion methods decreased the average percentage of host-specific reads according to the data obtained on both sequencing platforms, a statistically significant reduction was observed only in samples treated with the alternative DNase. Metataxonomic analysis of the abundant reads confirmed a reduction of most targeted bacterial reads during depletion and a consequent rise of untargeted bacterial, viral, and human reads. The increase of targeted reads belonging to family *Enterobacteriaceae* in treated samples may be attributed to potential contamination from polymerases and other enzymes, which are known source of *Escherichia coli* contamination (Koponen et al., 2002; Silkie et al., 2008; Stinson et al., 2019). This reduction in abundant host and bacterial rRNAs was instrumental in facilitating a notable increase in the number of sequenced influenza viral reads, contributing to enhanced breadth of viral genome coverage after consensus assembly. Similarly, to the host rRNA reduction results, the improvement in the breadth of viral genome was also statistically significant only in samples treated with the alternative DNase. The observed correlation between the reduction of host rRNA levels and the subsequent improvement in viral genome breadth highlights the potential utility of the RT-qPCR 28S-test in guiding further refinements of host rRNA depletion methods for enhanced detection of avian pathogens.

Furthermore, we conducted a thorough evaluation of the developed RT-qPCR 28S-test to monitor the effectiveness of different host depletion methods. In swab samples spiked with low pathogenic NDV, the comparison between DNase I and alternative DNase demonstrated the superior performance of the alternative DNase as well. Both methods significantly reduced host rRNA levels, as evidenced by elevated Ct values in the 28S-test. However, the alternative DNase outperformed DNase I, resulting in higher Ct values in the 28S-test, indicating more effective host rRNA reduction, and lower Ct values in the NDV M-test, signifying the preservation of viral RNA integrity. Thus, the alternative DNase was selected for further optimization of the depletion protocol due to its superior performance. Additionally, we explored cost-effective measures by utilizing a reduced set of probes (chicken 28S rRNA4 and rRNA5) instead of a complete probe set, which performed comparably to the complete set in the 28S-test. Also, we examined the potential use of rapid DNase to reduce preparation time. While rapid DNase offered a quicker incubation period (2 min) compared to DNase I (30 min) and the alternative DNase (30 min), it exhibited lower efficacy in host rRNA reduction compared to DNase I and the alternative DNase. This emphasizes the importance of balancing efficiency and effectiveness in depletion protocols.

We also investigated filtration pre-treatment using different syringe filters (0.45 μm Nylon, 0.45 μm Nalgene SFCA, and 0.22 μm Nalgene SFCA) before nucleic acid extraction. Comparing the three different filters among themselves, we observed no significant differences in the reduction of 28S chicken rRNA, regardless of whether post-extraction alternative DNase depletion treatment was applied or not. However, when compared to untreated samples, only the filtration with cellulose acetate filters led to a significant reduction in host rRNA levels when used for pre-extraction treatment without subsequent alternative DNase depletion. However, when combined with the depletion treatment, all extraction pre-treatments significantly reduced host rRNA compared to untreated samples. Nevertheless, these reductions were more pronounced than those achieved when the filters were used without following alternative DNase treatment. Though filtration combined with the alternative DNase effectively reduced host rRNA, it significantly decreased viral RNA as well. In contrast, the alternative DNase without filtration pre-treatment maintained host rRNA reduction while preserving viral RNA. The primary rationale behind using filtration was to selectively remove host and bacterial cells, with the expectation that virus particles, being significantly smaller, would pass through the filter and be present in higher concentrations and allow a higher percentage of viral sequencing reads. However, our unexpected outcome suggests two plausible explanations for the observed significant reduction of the viral RNA. The most likely explanation is that the virus particles are attached to host cells through receptor mediated interaction, and these virus/host cells were then removed by the filters. Second, variations in viral sizes may have played a role. For instance, orthomyxo- and paramyxoviruses are known to be pleomorphic and capable of producing virions of different shapes (Donald and Isaacs, 1954; Badham and Rossman, 2016). Generally, NDV virions are rounded and 100–500 nm in diameter, although filamentous forms of about 100 nm across and of variable length are often seen (Alexander and Senne, 2008; Goff et al., 2012; Miller and Koch, 2013; Burrell et al., 2017). Clinical isolates of influenza A virus have been shown to produce elongated filamentous particles up to 30 μm, whereas laboratory-adapted strains are predominantly spherical ranging from 80 to 120 nm (Cox et al., 1980; Roberts et al., 1998; Vijayakrishnan et al., 2013; Dadonaite et al., 2016; Leyson et al., 2021). Therefore, while filtration is a widely used technique for separating viruses from bacterial and host cells, our findings align with a prior observation (Conceição-Neto et al., 2015; Burke et al., 2019) and emphasize the importance of carefully considering filter pore size and material. Because of the large reduction in viral RNA as measured by RT-qPCR, we did not evaluate the filtered samples by sequence analysis, and additional investigation is needed to evaluate if there is a positive effect from filtering in increasing viral RNA reads.

Finally, our study explored various experimental parameters to optimize the depletion protocol’s performance. These investigations included extending the alternative DNase digestion time, testing different concentrations of the partial probe set, and conducting repeated RNase H treatments. Extending the alternative DNase digestion time beyond the recommended 30 min did not significantly impact the reduction of host rRNA or viral RNA levels. Similarly, varying the concentrations of the partial probe set did not yield significant differences in the 28S-test when probe concentrations were increased. When assessing repeated RNase H treatments to enhance the degradation of probe-bound rRNAs, one and three rounds of RNase H treatment resulted in a significant reduction in host rRNA levels compared to untreated samples. However, no statistically significant difference was observed when depletion was performed without RNase H treatment or with two rounds of RNase H treatment.

In summary, our comprehensive assessment of different depletion treatments with a specific emphasis on the performance evaluation of the 28S rRNA RT-qPCR, provides valuable insights to enhance avian virus detection through NGS. Careful selection of depletion methods, probe sets, and filtration pre-treatment steps is crucial for optimizing NGS outcomes. Therefore, this systematic optimization process ensures that our custom RNase H-based depletion protocol is fine-tuned for effective reduction of the most abundant host and bacterial rRNA, ultimately improving the detection, number of sequenced reads, and genome coverage for various avian viruses. The results presented in this study offer significant findings for further refining depletion methods and their influence on NGS performance in avian disease research.

## 5 Conclusion

In conclusion, our study highlights the effectiveness of the developed 28S rRNA RT-qPCR in evaluating host depletion methods for NGS detection of avian viruses. This assay facilitated the optimization of our established depletion protocol and the evaluation of other depletion methods. Substituting DNase I with the alternative DNase in our established depletion protocol resulted in improved outcomes, as validated by NGS data. Ultimately, our refined protocol, utilizing the alternative DNase, proved to be the overall optimal depletion method when compared to other DNases or filtration pre-treatments. The 28S-test provides a valuable foundation for the development and further refinement of host depletion strategies, ultimately enhancing the sensitivity and accuracy of avian virus detection in clinical samples. Additional investigations into optimizing depletion methods and their application in avian virology are warranted to advance our understanding of avian diseases and improve surveillance and control measures.

## Data availability statement

The original contributions presented in the study are publicly available. This data can be found at: NCBI Sequencing Read Archive (SRA) under BioProject Number PRJNA1021187.

## Ethics statement

The animal study protocol was approved by the Institutional Laboratory Animal Care and Use Committee of the United States National Poultry Research Center, ARS, USDA.

## Author contributions

IG: Conceptualization, Data curation, Formal analysis, Investigation, Methodology, Software, Validation, Visualization, Writing – original draft. MH: Formal analysis, Investigation, Methodology, Software, Validation, Writing – review & editing. ES: Funding acquisition, Methodology, Resources, Supervision, Writing – review & editing. DS: Conceptualization, Data curation, Formal analysis, Funding acquisition, Methodology, Project administration, Supervision, Writing – review & editing.
