# Supplementary material for: The 28S rRNA RT-qPCR assay for host depletion evaluation to enhance avian virus detection in Illumina and Nanopore sequencing
Source: Front Microbiol. 2024 Jan 31;15:1328987. doi: 10.3389/fmicb.2024.1328987 (PMC10864109; doi:10.3389/fmicb.2024.1328987)
Supplement: Supplementary file 1 [file Table_1.DOCX]

| **Treatment** | **Sample** | **SEPRL ID** | **RT-qPCR, Ct** | |  | **Nanopore** | | | | |  | **Illumina** | | | | |
| --- | --- | --- | --- | --- | --- | --- | --- | --- | --- | --- | --- | --- | --- | --- | --- | --- |
|  |  |  | **28S** | **AIV** |  | **Total Reads** | **Mapped Host Reads** | **Kraken Bacterial Reads** | **Mapped AIV Reads** | **AIV Genome Coverage, %** |  | **Total Reads** | **Mapped Host Reads** | **Kraken Bacterial Reads** | **Mapped AIV Reads** | **AIV Genome Coverage, %** |
| **Untreated** | 1 | 3627_a | 21.2 | 20.2 |  | 175,692 | 42671 | 128,587 | 2,201 | 98 |  | 542,314 | 106,515 | 420,980 | 9,674 | 99 |
|  | 2 | 3631_a | 16.8 | 23.7 |  | 79,896 | 23102 | 53,746 | 32 | 2 |  | 408,842 | 56,512 | 340,518 | 32 | 3 |
|  | 3 | 3632_a | 14.3 | 21.5 |  | 169,855 | 89,365 | 77,861 | 44 | 11 |  | 551,192 | 213,110 | 326,580 | 46 | 5 |
|  | 4 | 3634_a | 16.9 | 19.8 |  | 157,787 | 107,646 | 43,898 | 2,102 | 97 |  | 548,290 | 289,387 | 222,760 | 7,248 | 97 |
|  | 5 | 3635_a | 16.7 | 22.1 |  | 536,662 | 224,323 | 306,370 | 257 | 51 |  | 771,914 | 182,750 | 584,910 | 134 | 18 |
|  | **Average** |  | **17.2** | **21.5** |  | **223,978** | **97,421** | **122,092** | **927** | **52** |  | **564,510** | **169,655** | **379,150** | **3,427** | **44** |
| **DNase I** | 1 | 3627_b | 29.1 | 21.5 |  | 196,039 | 72,357 | 96,360 | 6,045 | 93 |  | 320,376 | 48,209 | 76,772 | 7,124 | 88 |
|  | 2 | 3631_b | 27.5 | 24.7 |  | 124,587 | 36,295 | 68,286 | 236 | 56 |  | 600,540 | 68,713 | 178,560 | 474 | 59 |
|  | 3 | 3632_b | 27.4 | 25.5 |  | 138,837 | 51,242 | 66,904 | 871 | 85 |  | 362,250 | 55,912 | 97,492 | 1,348 | 85 |
|  | 4 | 3634_b | 27.9 | 23.9 |  | 230,322 | 91,799 | 116,265 | 2,313 | 88 |  | 436,574 | 78,399 | 138,660 | 3,208 | 80 |
|  | 5 | 3635_b | 28.0 | 25.8 |  | 280,950 | 98,675 | 161,348 | 1,153 | 84 |  | 532,772 | 96,236 | 240,074 | 1,700 | 78 |
|  | **Average** |  | **28.0** | **24.3** |  | **194,147** | **70,074** | **101,833** | **2,124** | **81** |  | **450,502** | **69,494** | **146,312** | **2,771** | **78** |
| **Alternative DNase** | 1 | 3627_c | 35.2 | 20.9 |  | 59,218 | 5,836 | 7,167 | 13,532 | 98 |  | 543,946 | 42,482 | 24,294 | 26,232 | 96 |
|  | 2 | 3631_c | 33.5 | 24.2 |  | 84,544 | 10,070 | 50,718 | 466 | 73 |  | 606,892 | 48,624 | 183,962 | 1,074 | 77 |
|  | 3 | 3632_c | 32.9 | 24.3 |  | 134,724 | 50,438 | 61,283 | 2,794 | 91 |  | 525,020 | 87,657 | 159,406 | 4,612 | 92 |
|  | 4 | 3634_c | 33.1 | 22.9 |  | 70,841 | 18,364 | 10,707 | 19,230 | 98 |  | 494,620 | 53,800 | 35,966 | 42,378 | 97 |
|  | 5 | 3635_c | 31.4 | 25.2 |  | 185,965 | 37,317 | 120,103 | 5,190 | 94 |  | 470,064 | 55,625 | 231,932 | 7,082 | 91 |
|  | **Average** |  | **33.2** | **23.5** |  | **107,058** | **24,405** | **49,996** | **8,242** | **91** |  | **528,108** | **57,638** | **127,112** | **16,276** | **91** |

**Supplementary Table 1.** RT-qPCR and sequencing data for each sample in different depletion treatments.
