# Supplementary material for: The 28S rRNA RT-qPCR assay for host depletion evaluation to enhance avian virus detection in Illumina and Nanopore sequencing
Source: Front Microbiol. 2024 Jan 31;15:1328987. doi: 10.3389/fmicb.2024.1328987 (PMC10864109; doi:10.3389/fmicb.2024.1328987)
Supplement: Supplementary file 2 [file Image_1.PDF]

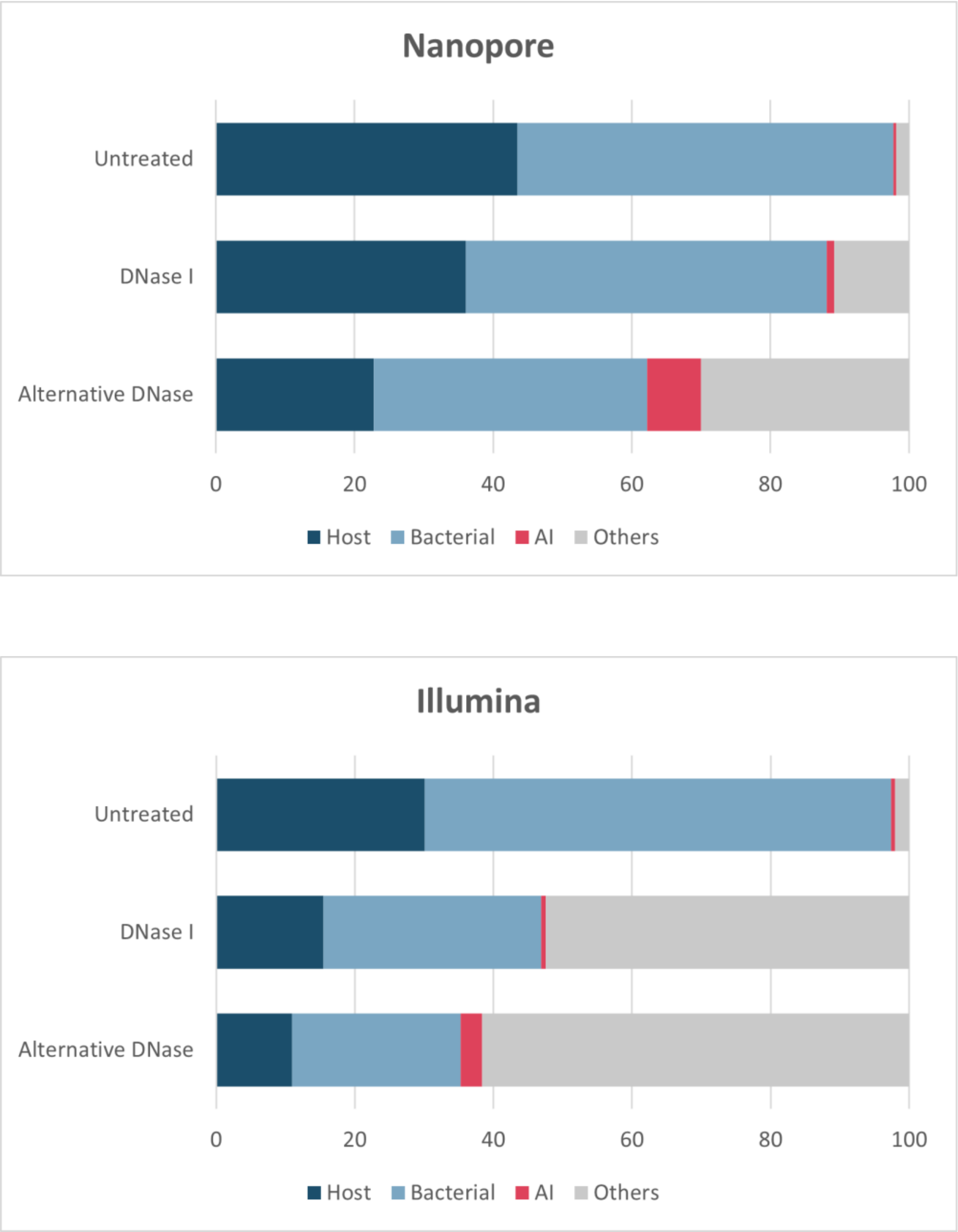

Supplementary Figure 1. Average percentage of host, bacterial, and avian influenza reads contribution obtained on **(A)** Nanopore and **(B)** Illumina sequencing platforms.
