# Supplementary material for: The 28S rRNA RT-qPCR assay for host depletion evaluation to enhance avian virus detection in Illumina and Nanopore sequencing
Source: Front Microbiol. 2024 Jan 31;15:1328987. doi: 10.3389/fmicb.2024.1328987 (PMC10864109; doi:10.3389/fmicb.2024.1328987)
Supplement: Supplementary file 3 [file Image_2.PDF]

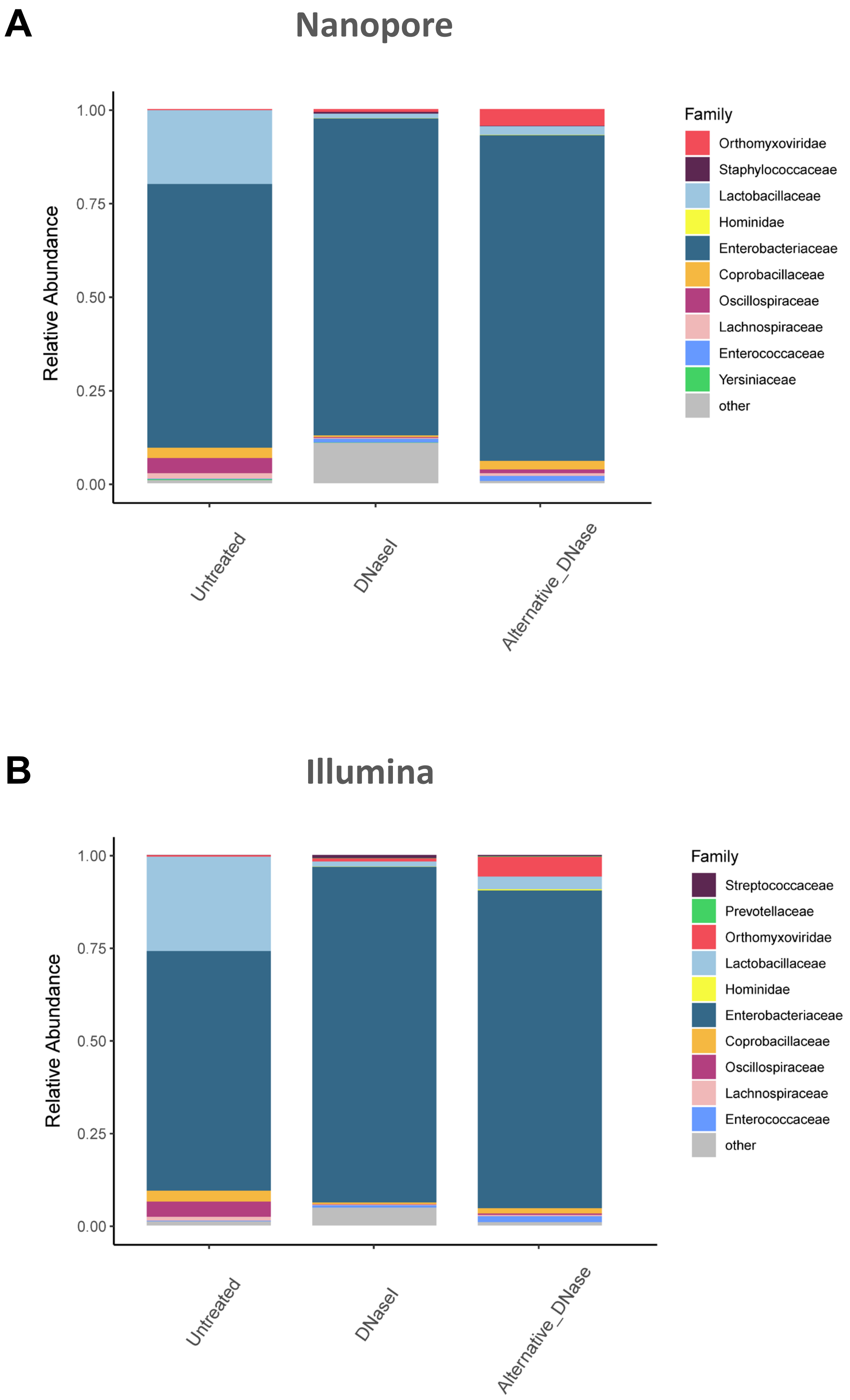

Supplementary Figure 2. Relative abundance of non-host reads at the family level as estimated by Kraken2/Bracken obtained on **(A)** Nanopore and **(B)** Illumina sequencing platforms. Only a subsample of 10 taxa with the greatest median relative abundances from the total community are shown.
