# Supplementary material for: The 28S rRNA RT-qPCR assay for host depletion evaluation to enhance avian virus detection in Illumina and Nanopore sequencing
Source: Front Microbiol. 2024 Jan 31;15:1328987. doi: 10.3389/fmicb.2024.1328987 (PMC10864109; doi:10.3389/fmicb.2024.1328987)
Supplement: Supplementary file 4 [file Image_3.PDF]

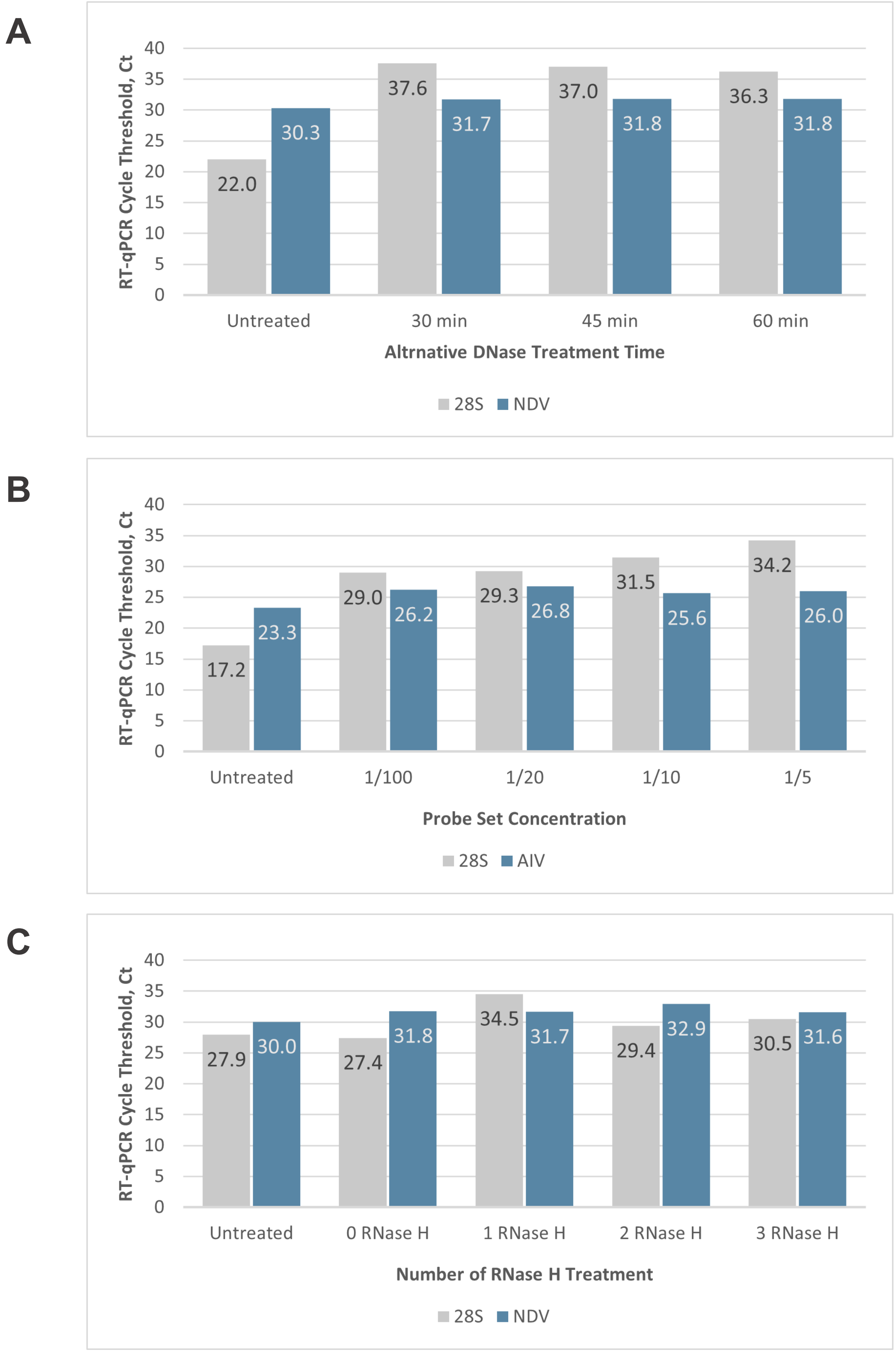

Supplementary Figure 3. Average host and viral RT-qPCR cycle threshold (Ct) values after different **(A)** alternative DNase digestion time (30, 45, and 60 minutes); **(B)** alternative DNase depletion treatment with different concentrations of partial hybridization probe set; **(C)** alternative DNase depletion treatment with different number of RNase H digestions. Depletion with one RNase H digestion provided a higher reduction of host 28S rRNA. However, prolonged Turbo DNase digestion did not significantly impact the reduction of 28S rRNA and viral RNA compared to recommended 30-minute digestion. Partial probe concentrations showed a common trend of an increased Ct value targeting 28s rRNA when concentrations increased. Viral Ct values were not influenced by partial probe concentration between groups.
